# Euthanasia and assisted suicide in people with mental disorders: a case report

**DOI:** 10.1192/j.eurpsy.2024.1196

**Published:** 2024-08-27

**Authors:** M. Martin De Argila Lorente, B. Franco Lovaco, M. González Guembe, M. Fresnillo Palomera, C. Díaz Mayoral

**Affiliations:** ^1^Psychiatry, Hospital Dr. Rodríguez Lafora; ^2^Psychiatry, Hospital Universitario Príncipe de Asturias, Madrid, Spain

## Abstract

**Introduction:**

Until 2020, only Belgium, Luxembourg, Switzerland, and the Netherlands legalized euthanasia and assisted suicide in Europe. Spain joined this list in March 2021 with the Euthanasia Regulation Law. However, the practice of euthanasia and assisted suicide in individuals with severe mental disorders is complex due to potential cognitive and decision-making challenges. Psychiatrists play a vital role in evaluating such requests.

**Objectives:**

he case of a patient with recurrent depressive disorder requesting euthanasia is presented, followed by a theoretical review of the subject.

**Methods:**

A case is presented with a bibliographic review.

**Results:**

An 89-year-old man with a history of one prior brief psychiatric hospitalization for depression three years ago was admitted after attempting suicide with an overdose of medication. He reports depressive symptoms of several years of evolution. Medical tests came back normal, but he had a urinary catheter due to voiding issues. He was initially on a medication regimen of amitriptyline, clomethiazole, and fluvoxamine. Despite his depressive state, he maintained his cognitive and decision-making abilities. Medication adjustments were made, including discontinuing amitriptyline and switching fluvoxamine to amitriptyline. His depressive symptoms worsened after three days, leading to the addition of trazodone to his treatment. He also developed urinary symptoms and was diagnosed with a urinary tract infection and metastatic prostate cancer during urological evaluation. Emotionally, he became more apathetic, anergic, and anhedonic, frequently expressing a desire for euthanasia, even with medication changes. Hyponatremia led to the discontinuation of duloxetine and the introduction of venlafaxine. To address anxiety and sleep problems, clomethiazole was replaced with mirtazapine. Upon learning of his cancer diagnosis, his mood deteriorated further, along with increased anxiety and continued mentions of euthanasia. Lorazepam was introduced, and he was informed of his right to request euthanasia after discussing therapeutic options with urology. Following this consultation, the patient became calmer, stopped expressing thoughts of death, and began making short-term plans, including the possibility of receiving palliative care at home upon discharge.

**Conclusions:**

Euthanasia and assisted suicide in severe mental disorders are complex due to ethical and medical challenges. Patients must understand their condition, prognosis, and have decision-making capacity. Assessing their suffering is crucial. Coexisting mental and organic issues complicate the request’s origin. In SMD, determining irreversibility is tricky, as these are often chronic, non-terminal conditions. Exhausting treatment options is essential before considering euthanasia, despite patient treatment refusal. Limited research underscores the need for more studies.

**Disclosure of Interest:**

None Declared

